# Magnetization transfer explains most of the $T_{1}$ variability in the MRI literature

**Published:** 2024-09-09

**Authors:** Jakob Assländer

**Affiliations:** 1 Center for Biomedical Imaging, Dept. of Radiology, NYU School of Medicine, NY, USA; 2 Center for Advanced Imaging Innovation and Research (CAI^2^R), Dept. of Radiology, NYU School of Medicine, NY, USA

**Keywords:** T1, magnetization transfer, MT, relaxometry, quantitative MRI, parameter mapping

## Abstract

**Purpose:**

To identify the predominant source of the $T_{1}$ variability described in the literature, which ranges from 0.6–1.1s for brain white matter at 3T.

**Methods:**

25 $T_{1}$-mapping methods from the literature were simulated with a mono-exponential and magnetization-transfer (MT) models, each followed by mono-exponential fitting. A single set of model parameters was assumed for the simulation of all methods, and these parameters were estimated by fitting the simulation-based to the corresponding literature $T_{1}$ values of white matter at 3T.

**Results:**

Mono-exponential simulations suggest good inter-method reproducibility and fail to explain the highly variable $T_{1}$ estimates in the literature. In contrast, MT simulations suggest that a mono-exponential fit results in a variable $T_{1}$ and explain up to 62% of the literature’s variability.

**Conclusion:**

The results suggest that a mono-exponential model does not adequately describe longitudinal relaxation in biological tissue. Therefore, $T_{1}$ in biological tissue should be considered only a *semi-quantitative* metric that is inherently contingent upon the imaging methodology; and comparisons between different $T_{1}$-mapping methods and the use of simplistic spin systems—such as doped-water phantoms—for validation should be viewed with caution.

## 1 INTRODUCTION

The Bloch equations^1^ are the bedrock for our understanding of magnetic resonance imaging (MRI). They are governed by two time constants, $T_{1}$ and $T_{2}$, which characterize the relaxation of longitudinal and transverse magnetization, respectively. Clinical MRI protocols rely on spin relaxation in the form of $T_{1}$- and $T_{2}$-*weighted* images. Quantification of these parameters, which has been desired since MRI’s inception, promises a more objective assessment of the biochemical environment of tissue and the hypothesis that $T_{1}$ and $T_{2}$ are quantitative biomarkers motivates their use in large, multi-center studies and artificial intelligence. However, the widespread adoption of quantitative relaxometry has been hampered by long scan times and considerable variability in parameter estimates, particularly for $T_{1}$ where the range is 0.6–1.1s for brain white matter at 3T.^2–5^ While scan times have been progressively reduced,^6–9^ $T_{1}$ variability remains a key challenge and decades of research have failed to provide a consensus $T_{1}$ mapping method.

Numerous explanations for this variability have been hypothesized, including inhomogeneities of the radio frequency (RF) field ($B_{1}^{+}$),^2^ incomplete RF spoiling,^2^ and magnetization transfer (MT).^3,5,10,11^ This paper identifies MT, i.e., the interaction between spins associated with liquids and macromolecules,^12,13^ as the dominant cause, which has profound implications for our understanding of spin relaxation. While mono-exponential relaxation, which is ingrained in the Bloch equations, has a theoretical underpinning for pure liquids,^14^ it does not accurately characterize the spin dynamics in biological tissues, resulting in considerable dependency of $T_{1}$ estimates on the imaging method.

Previous studies analyzed MT in individual $T_{1}$-mapping methods.^3,5,15^ This study analyses a representative set of the prevalent methods in the literature and demonstrates that MT explains 62% of the reported $T_{1}$ variability. The best results are achieved when incorporating two recent advances in our understanding of MT: the discovery that the $T_{1}$ of different spin pools differ substantially^10,16,17^ and that RF pulses rotate the magnetization of the macromolecular pool rather than saturate it, as described by the generalized Bloch model.^18^ This result suggests that $T_{1}$ in biological tissue should be considered only a *semi-quantitative* metric. The following sections discuss implications for the interpretation of past $T_{1}$ mapping studies and provide suggestions for future directions, including measures for improved inter-study comparability and avenues toward developing methods for fully quantitative biomarkers.

## 2 METHODS

This study focuses on $T_{1}$ mapping of brain white matter at 3T, for which 25 methods were selected from the literature, including different implementations of inversionrecovery,^2,15,19–23^ Look-Locker,^2,21^ saturation-recovery,^15^ variable flip angle,^2,5,20,24–26^ MP-RAGE,^23^ and MP_2_RAGE.^27^ Different implementations of the same techniques vary in shape, amplitude, and timing of RF pulses. The signal of each method was simulated with various MT models with an emphasis on capturing the RF scheme adequately, while neglecting imaging gradients and assuming complete RF spoiling as well as homogeneous $B_{0}$ and $B_{1}^{+}$ fields. Sequence details such as timing, RF pulse shapes and amplitudes were extracted from the publications and complemented with information kindly provided by authors in private communications. Missing information was filled by educated guesses and the source of information for each sequence detail are denoted in the publicly available simulation code (cf. Appendix B). Sequence-specific $T_{1}$ values were estimated from the simulated data of each pulse sequence with the fitting procedures described in the respective publication.

All pulse sequences were simulated with a global set of relaxation times and MT parameters. Considering all $T_{1}$-mapping methods jointly, least-squares fitting was used to estimate these parameters to best explain the $T_{1}$ variability.

This procedure was repeated with 4 models: a mono-exponential model, Graham’s spectral MT model,^18,28^ and the generalized Bloch MT model.^18^ The latter was simulated twice, once with the commonly-used constraint $T_{1}^{s}=T_{1}^{f}$, i.e., assuming equal relaxation times for both pools and once without this constraint. Graham’s spectral MT model was simulated with an unconstrained $T_{1}^{s}$. Further, the transversal relaxation times and the exchange rate were fixed in all fits to ensure fit stability (Tab. 1).

The saturation of the semi-solid spin pool was simulated during all RF pulses, including inversion, excitation, and refocussing pulses. Since many pulses are on-resonant, Graham’s spectral model was used rather than the more common Graham’s single-frequency approximation. As described in Ref. 18, the former is an intermediate step in Graham’s original publication,^28^ which takes the integral over the line shape, multiplied by the RF pulse’s power spectral density. This approach integrates of the singularity of the super-Lorentzian line shape, which is well-defined and numerically stable.

## 3 RESULTS

**FIGURE 1:**
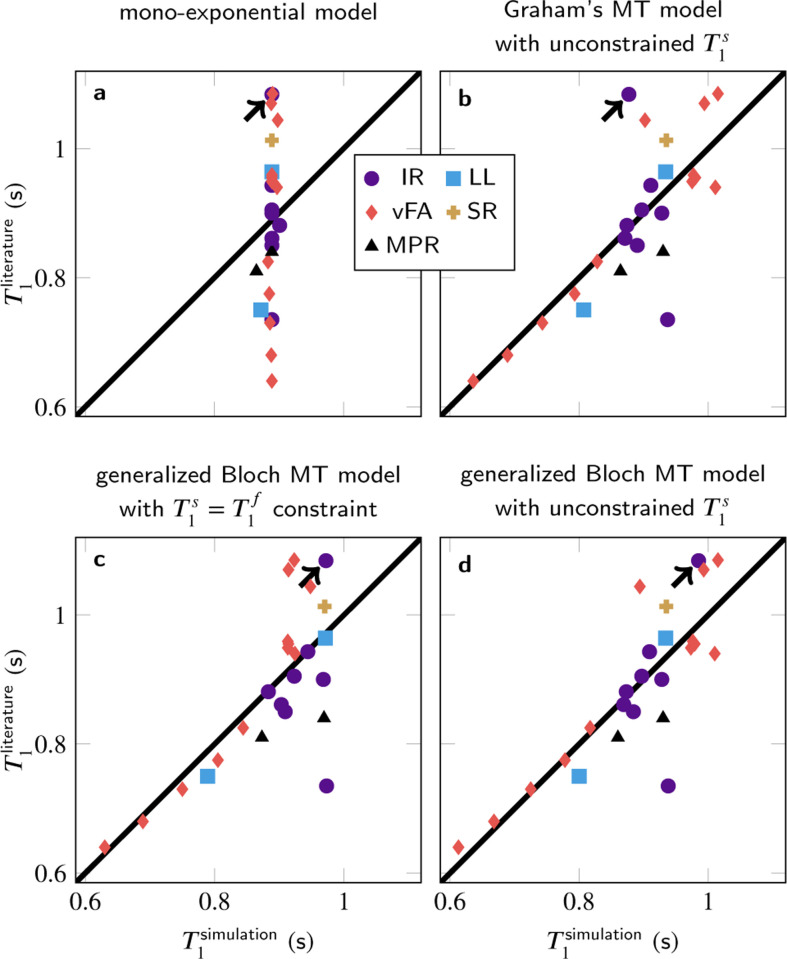
Literature $T_{1}$ estimates based on measured data in comparison to $T_{1}$ estimates from MT simulations. Overall, 25 $T_{1}$-mapping methods were simulated, comprising different implementations of inversion-recovery (IR), Look-Locker (LL), variable flip angle (vFA), saturation-recovery (SR), and MP_(2)_RAGE (MPR). The arrows highlight an IR method with a very short inversion pulse.

In contrast to the 3% intra-study coefficients of variation reported for $T_{1}$,^29^ the inter-study coefficient of variation is 14% across the literature analyzed here. Fig. 1 illustrates this variability by the spread along the y-axis, and compares it to $T_{1}$ estimates based on signals that were simulated for respective data acquisition method, followed by mono-exponential fitting as described in the respective publication. The simulations of all acquisition methods used a global set of model parameters, which was determined with a least-square fitting procedure to best explain the literature $T_{1}$ values (Tab. 1).

Simulating the signals with a mono-exponential model (Fig. 1a) results in a small span along the x-axis, indicating inter-study reproducibility within the mono-exponential framework, which matches experimental findings in phantoms containing doped water.^2^ However, the deviations from the identity line indicate that a mono-exponential model fails to explain the inter-study variability observed in tissue.

Simulating the signal with various MT models and fitting a mono-exponential model to the simulated data replicates most of the $T_{1}$ variability (b–d), i.e., the median absolute deviation is reduced by 62% when comparing the residuals of the generalized Bloch fit without $T_{1}^{s}$ constraint to the $T_{1}$ estimates in the literature. To provide some context for this result, note that the simulations are based on incomplete knowledge of implementation details, despite many authors kindly providing unpublished information. Incorrect implementation details can result in outliers, which were not excluded from the least-square fitting of the MT parameters or any other analysis. Outliers impair the performance of least-square fitting, which intrinsically assumes a Gaussian distribution of residuals. As the residuals’ distribution is unknown, least-squares fitting is used for simplicity, and to ensure a stable fit, literature values were used for the transversal relaxation times and the exchange rate. The other MT parameters were fitted and align well with the literature (Tab. 1). Removing all constraints further reduces the residuals, at the cost of less plausible MT parameters.

Different MT models capture the $T_{1}$ variability to slightly different degrees: Graham’s spectral model^28^ does not adequately describe the spin dynamics during a 10μs inversion-pulse (arrow in Fig. 1b). This challenge is overcome by the generalized Bloch model^18^ (c–d). Further, the commonly-used constraint $T_{1}^{s}=T_{1}^{f}$ (where the superscripts denote the semi-solid or macromolecular and free pool, respectively) entails larger residuals compared to the recently proposed unconstrained fit (c vs. d). The Akaike and Bayesian information criteria (Tab. 2) indicate that a fit with the generalized Bloch model and without $T_{1}^{s}$ constraint best explains the $T_{1}$ variability and that the increased number of variables is justified.

## 4 DISCUSSION

Only one year after the discovery of MT,^12^ Koenig et al.^31^ hypothesized an association between MT and $T_{1}$ relaxation. Notwithstanding, MT has traditionally been considered a nuisance effect in $T_{1}$ mapping and, likely due to time constraints of *in vivo* imaging settings, most methods assume a mono-exponential model. Recent studies, however, picked up on Koenig’s hypothesis and suggest that MT is an integral driver of longitudinal relaxation.^10,16,17^ This paper analyzes the variability in mono-exponential $T_{1}$ estimates throughout the literature and links it to pervasive but variable contributions of MT.

In the absence of RF pulses, e.g., during an inversion-recovery experiment, the two-pool MT model describes biexponential relaxation.^13,32^ Fitting a mono-exponential model to such data elicits a sensitivity of the estimated $T_{1}$ to the inversion times, explaining the observed variability. This brings into question the common classification of the inversion-recovery method with mono-exponential fitting as the gold standard for $T_{1}$ mapping in biological tissue.

RF pulses affect the two spin pools differently due to their vastly different $T_{2}$ relaxation times (10μs vs. 100ms). As a consequence, the measured signal is sensitive to the shape and amplitude of the RF pulses, as well as the timing of their sequence. This sensitivity includes inversion-recovery methods and is pronounced for variable flip angle methods, which rely on many RF pulses in rapid succession.

The finding that MT explains most of the $T_{1}$ variability indicates that the principal cause is an oversimplified model rather than experimental limitations, which positions $T_{1}$ in biological tissue as a *semi-quantitative* metric, inherently contingent upon the employed imaging methodology. It questions the comparability of different $T_{1}$-mapping techniques and suggests that validations conducted in simplistic spin systems, such as doped-water phantoms, might provide only a partial assessment of $T_{1}$-mapping methods.

It is important to note that different imaging methods do not result in re-scaled versions of the same $T_{1}$. On the contrary, different methods capture different weightings of the individual relaxation mechanisms and might have different sensitivities to pathology, making them fundamentally incomparable. Notably, even small variations in the data acquisition protocol can influence the contributions of different relaxation mechanisms as exemplified by the inversion-recovery method: short inversion times are sensitive to the exchange rate, while long inversion times are mostly sensitive to the spin-pool size $m_{0}^{s}$ and the pools’ relaxation times $T_{1}^{f,s}$.^17,32^ For most methods, however, the composition of relaxation mechanisms is not intuitively evident.

One path toward more reproducible $T_{1}$ mapping would be to design methods in which each data point has a similar sensitivity to the MT parameters. For inversion-recovery methods, this could be achieved by acquiring data only at inversion times much longer than the fast component, i.e., much longer than 100ms.^17,32^ For variable flip angle methods, Teixeira et al.^5^ suggested adding off-resonant saturation to each RF pulse such that the macromolecular spin pool is kept constant over variable flip angles. The resulting relaxation model is mono-exponential with a composition of relaxation mechanisms that depends on the applied RF power. Teixeira et al. proposed to further qualify the reported $T_{1}$ values by the applied RF power to identify studies that assess similar compositions.

This paper provides a comprehensive comparison of established $T_{1}$-mapping methods and identifies the relaxation model as the principal bottleneck on the road to quantitative biomarkers. The presented findings suggest that a separation of the individual relaxation mechanisms, as performed in quantitative MT, is necessary to quantify longitudinal relaxation without major dependencies on implementation details. However, it is noted that any model entails simplifications, especially considering the complexity of biological tissue. Further research is needed to identify adequate compromises between model complexity and method-dependent bias.

## Figures and Tables

**TABLE 1:** Estimates of MT parameters. The parameters were estimated by fitting MT models to variable literature $T_{1}$ values (*this*) and are here compared to MT parameters reported in the literature.^10,17,19,30^ $m_{0}^{s}$ denotes the macromolecular or semi-solid spin pool size, the relaxation times $T_{1,2}$ are qualified by the superscripts ^*f, s*^ to identify the free and macromolecular or semi-solid spin pool, respectively, and $R_{x}$ is the exchange rate. The gray background highlights parameters that were fixed during the fit.

MT model	Graham’s		generalized Bloch			

$T_{1}^{s}$ constraint	none		$T_{1}^{s}=T_{1}^{f}$		none	

study	this	^10,19^	this	^19,30^	this	^17^
$m_{0}^{s}$	0.19	0.27	0.13	0.14	0.21	0.21
$T_{1}^{f}$ (s)	2.03	2.44	0.97	1.52	2.06	1.84
$T_{1}^{s}$ (s)	0.25	0.25	$T_{1}^{f}$		0.26	0.34
$T_{2}^{f}$ (ms)	76.9	69	76.9	70.1	76.9	76.9
$T_{2}^{s}$ (μs)	12.5	10.0	12.5		12.5	12.5
$R_{x}$ (s^−1^)	13.6	9.0	23.0	23.0	13.6	13.6

**TABLE 2:** Akaike (AIC) and Bayesian (BIC) information criteria. The values are relative to the mono-exponential fit ($\text{ΔAIC}=\text{AIC}-{\text{AIC}}_{\text{mono}}$) and lower values indicate a preferable model. AIC and BIC weigh residuals against the number of model parameters and the results indicate that the generalized Bloch model without $T_{1}^{s}$ constraint is preferable despite the penalty for its larger number of parameters.

model	$T_{1}^{s}$ constraint	ΔAIC	ΔBIC
mono-exponential	none	0	0
Graham’s	none	−18.6	−16.1
generalized Bloch	$T_{1}^{s}=T_{1}^{f}$	−16.8	−15.6
generalized Bloch	none	−24.7	−22.3

## Data Availability

Code to replicate all results can be found at https://jakobasslaender.github.io/T1variability/. This website outlines all simulation code along with the here-presented results. Contributions of additional $T_{1}$-mapping methods as well as improvements of the current methods are explicitly welcomed and can be facilitated via GitHub pull-requests. The fitting results are continuously updated with continuous-integration tools.
